# The genome sequence of the flat clown beetle, *Hololepta plana* (Sulzer, 1776) (Coleoptera: Histeridae)

**DOI:** 10.12688/wellcomeopenres.26588.1

**Published:** 2026-05-18

**Authors:** Michael F. Geiser, Jordan J. Rainey, Dmitry Telnov, Maxwell V. L. Barclay, Natural History Museum Genome Acquisition Lab, Darwin Tree of Life Barcoding Collective, Wellcome Sanger Institute Tree of Life Management, Samples and Laboratory team, Wellcome Sanger Institute Scientific Operations: Sequencing Operations, Wellcome Sanger Institute Tree of Life Core Informatics team, Tree of Life Core Informatics collective, Darwin Tree of Life Consortium

**Affiliations:** 1 Natural History Museum, London, England, UK; 2 Biology Centre, Czech Academy of Sciences, České Budějovice, Czech Republic; 3 Daugavpils University, Daugavpils, Latvia; 4 Institute of Biology, University of Latvia, Rīga, Latvia

**Keywords:** Hololepta plana, flat clown beetle, genome sequence, chromosomal, Coleoptera

## Abstract

We present a genome assembly from an individual female *Hololepta plana* (flat clown beetle; Arthropoda; Insecta; Coleoptera; Histeridae). The genome sequence has a total length of 139.51 megabases. Most of the assembly (97.93%) is scaffolded into 12 chromosomal pseudomolecules, including the X sex chromosome. The mitochondrial genome has also been assembled, with a length of 17.73 kilobases. This assembly was generated as part of the Darwin Tree of Life project, which produces genomes for eukaryotic species found in Britain and Ireland.

## Species taxonomy

Eukaryota; Opisthokonta; Metazoa; Eumetazoa; Bilateria; Protostomia; Ecdysozoa; Panarthropoda; Arthropoda; Mandibulata; Pancrustacea; Altocrustacea; Allotriocarida; Hexapoda; Insecta; Dicondylia; Pterygota; Neoptera; Eumetabola; Endopterygota; Aparaglossata; Neuropteroidea; Coleoptera; Polyphaga; Staphyliniformia; Histeroidea; Histeridae; Histerinae; Hololeptini; *Hololepta*; *Hololepta plana* (Sulzer, 1776) (NCBI:txid290657).

## Background

*Hololepta plana* (Sulzer, 1776) is a member of the family Histeridae, commonly known as the clown beetles. This species is the only representative of the tribe Hololeptini Hope, 1840 in the UK. The combined characters of large adult size (approximately 8.5 mm), a flattened body, and a reduced prosternal projection leaving the ventral surface of the head exposed make *H. plana* one of the most recognisable of the 54 histerid species in Britain ( Lane, 2017).

*Hololepta plana* is widely distributed across Europe and extends into Asia ( Lackner *et al.*, 2015). Sánchez-Ruiz *et al.* (2002) documented its range extending as far south as southern Spain. The northernmost known record is from Mäntsälä, Finland, reported by iNaturalist user “janijarvi” in 2022, with supporting images. The species is widespread in the boreal forest zone of Europe in the Baltic countries (D. Telnov, personal observations).

The species was first recorded in Britain from Norfolk in 2009 ( Allen & Hance, 2009). Additional records have been added since then, mostly from south-western England. Lane (2021) summarises the British records and Barclay (2018) expanded on this. It has been recorded in vice-counties including West Norfolk ( Allen & Hance, 2009; Collier & Lane, 2015; Lane *et al.*, 2021), Middlesex ( Barclay, 2018; Lane *et al.*, 2021; Telfer, 2014), West Suffolk ( Lane, 2017; Lane *et al.*, 2021), Cambridgeshire ( Lane *et al.*, 2021), Buckinghamshire and Surrey ( Lane *et al.*, 2021). Lane’s species status review ( Lane, 2017) gives the IUCN status of *H. plana* as Data Deficient and the GB Rarity Status as Nationally Rare. It seems likely that it is a recent immigrant in Britain, particularly given the complete absence of old records for such a conspicuous species.

Adults and larvae of *H. plana* prey on subcortical dipteran larvae ( Barclay, 2018; Trach, 2013). Their flattened bodies allow them to live beneath bark ( Kryzhanovsky & Reikhardt, 1976). In Britain, *H. plana* is most often found beneath the sappy, laminating bark of dead poplar trees ( *Populus* spp.) ( Lane, 2017). Barclay (2018) recorded the species from a dead tree of heaven ( *Ailanthus altissima*), which also supported a large population of subcortical dipteran larvae. Other recorded hosts include oak and pine ( *Quercus* sp. and *Pinus sylvestris*) and poplars including *Populus tremula* and *P. alba* ( Nagaraju *et al.*, 2022). In Latvia, the species has also been recorded beneath the bark of Norway maple ( *Acer platanoides*), although it appears to prefer European aspen ( *Populus tremula*) and other poplars (D. Telnov, unpublished data). Live specimens of *H. plana* were recently found from logs imported into India from Belgium ( Nagaraju *et al.*, 2022), suggesting that the species may have arrived in Britain via logs imported from mainland Europe.

**Figure 1.**
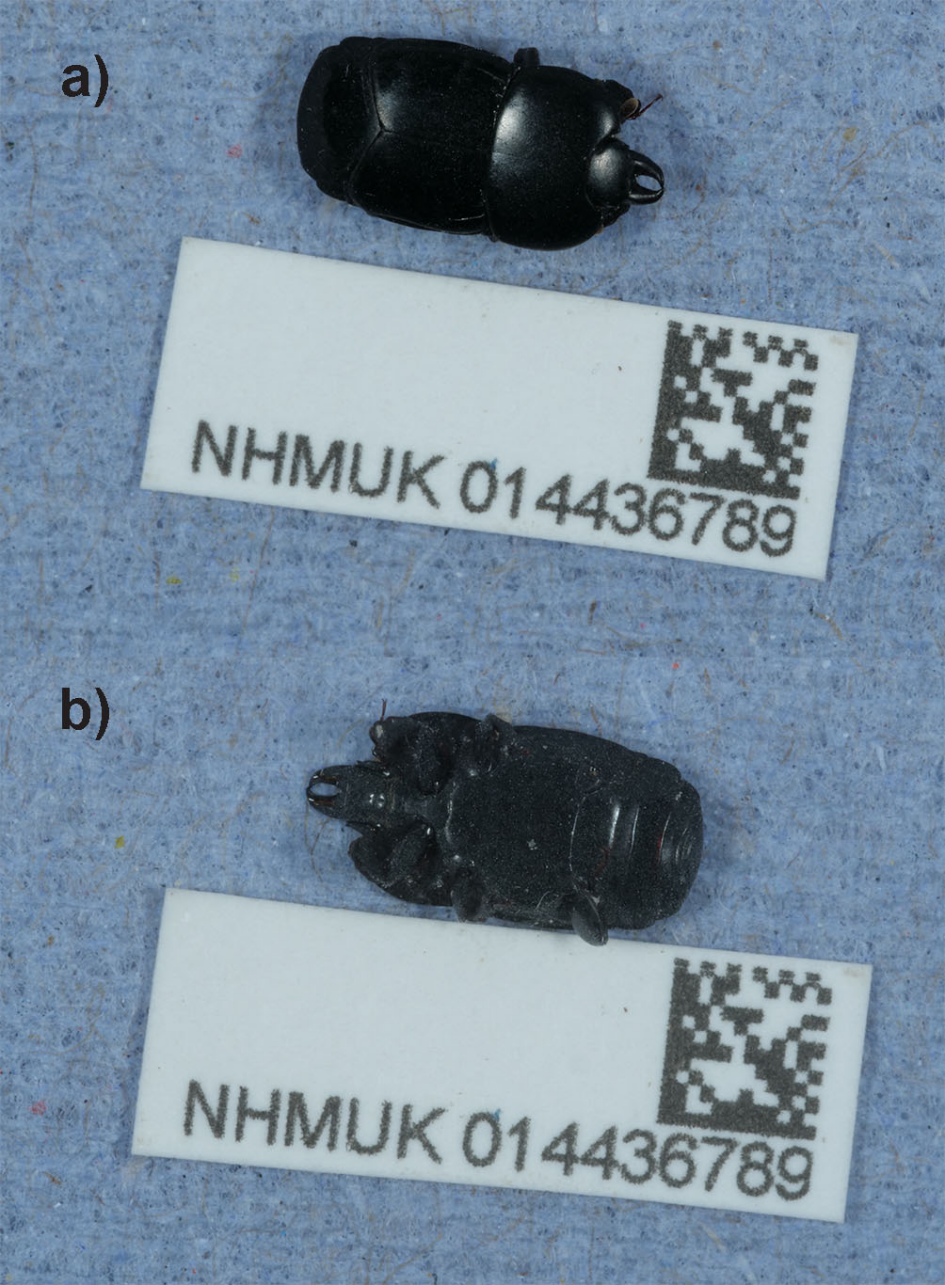
Photograph of the *Hololepta plana* (icHolPlan1) specimen used for genome sequencing.

A chromosome-level genome sequence for *H. plana* is presented here. The assembly was produced using the Tree of Life pipeline from a specimen collected in Middlesex, England ( Figure 1). The genome sequence will support investigations into the origin of the British population of *H. plana* and uncover likely connections with subpopulations of the species in continental Europe.

## Methods

### Sample acquisition and DNA barcoding

The specimen used for genome sequencing was an adult female *Hololepta plana* (specimen ID NHMUK014436789, ToLID icHolPlan1; Figure 1), collected from Wormwood Scrubs Park, Hammersmith And Fulham, Middlesex, England, United Kingdom (latitude 51.52, longitude −0.25) on 2021-07-17. The specimen was collected and identified by Michael Geiser (Natural History Museum).

The initial identification was verified by an additional DNA barcoding process according to the framework developed by Twyford *et al.* (2024). A small sample was dissected from the specimen and stored in ethanol, while the remaining parts were shipped on dry ice to the Wellcome Sanger Institute (WSI) (see the protocol). The tissue was lysed, the COI marker region was amplified by PCR, and amplicons were sequenced and compared to the BOLD database, confirming the species identification ( Crowley *et al.*, 2023). Following whole genome sequence generation, the relevant DNA barcode region was also used alongside the initial barcoding data for sample tracking at the WSI ( Twyford *et al.*, 2024). The standard operating procedures for Darwin Tree of Life barcoding are available on protocols.io.

### Nucleic acid extraction

Detailed protocols for nucleic acid extraction developed at the Wellcome Sanger Institute (WSI) Tree of Life Core Laboratory are available on protocols.io ( Howard *et al.*, 2025). The icHolPlan1 sample was weighed and triaged to determine the appropriate extraction protocol. Tissue from the head and thorax was homogenised by powermashing using a PowerMasher II tissue disruptor. High molecular weight (HMW) DNA was extracted using the Automated MagAttract v1 protocol. We used centrifuge-mediated fragmentation to produce DNA fragments in the 8–10 kb range, following the Covaris g-TUBE protocol for ultra-low input (ULI). Sheared DNA was purified by automated SPRI (solid-phase reversible immobilisation).

The concentration of the sheared and purified DNA was assessed using a Nanodrop spectrophotometer and Qubit Fluorometer using the Qubit dsDNA High Sensitivity Assay kit. Fragment size distribution was evaluated by running the sample on the FemtoPulse system. For this sample, the final post-shearing DNA had a Qubit concentration of 0.856 ng/μL and a yield of 333.84 ng.

### PacBio HiFi library preparation and sequencing

Library preparation and sequencing were performed at the WSI Scientific Operations core. Prior to library preparation, the DNA was fragmented to ~10 kb. Ultra-low-input (ULI) libraries were prepared using the PacBio SMRTbell® Express Template Prep Kit 2.0 and gDNA Sample Amplification Kit. Samples were normalised to 20 ng DNA. Single-strand overhang removal, DNA damage repair, and end-repair/A-tailing were performed according to the manufacturer’s instructions, followed by adapter ligation. A 0.85× pre-PCR clean-up was carried out with Promega ProNex beads.

The DNA was evenly divided into two aliquots for dual PCR (reactions A and B), both following the manufacturer’s protocol. A 0.85× post-PCR clean-up was performed with ProNex beads. DNA concentration was measured using a Qubit Fluorometer v4.0 (Thermo Fisher Scientific) with the Qubit HS Assay Kit, and fragment size was assessed on an Agilent Femto Pulse Automated Pulsed Field CE Instrument (Agilent Technologies) using the gDNA 55 kb BAC analysis kit. PCR reactions A and B were then pooled, ensuring a total mass of ≥500 ng in 47.4 μl.

The pooled sample underwent another round of DNA damage repair, end-repair/A-tailing, and hairpin adapter ligation. A 1× clean-up was performed with ProNex beads, followed by DNA quantification using the Qubit and fragment size analysis using the Agilent Femto Pulse. Size selection was performed on the Sage Sciences PippinHT system, with target fragment size determined by Femto Pulse analysis (typically 4–9 kb). Size-selected libraries were cleaned with 1.0× ProNex beads and normalised to 2 nM before sequencing.

The sample was sequenced using the Sequel IIe system (Pacific Biosciences, California, USA). The concentration of the library loaded onto the Sequel IIe was in the range 40–135 pM. The SMRT link software, a PacBio web-based end-to-end workflow manager, was used to set-up and monitor the run, and to perform primary and secondary analysis of the data upon completion.

### Hi-C

***Sample preparation and crosslinking***

The Hi-C sample was prepared from 20–50 mg of frozen abdomen tissue of the icHolPlan1 sample using the Arima-HiC v2 kit (Arima Genomics). Following the manufacturer’s instructions, tissue was fixed and DNA crosslinked using TC buffer to a final formaldehyde concentration of 2%. The tissue was homogenised using the Diagnocine Power Masher-II. Crosslinked DNA was digested with a restriction enzyme master mix, biotinylated, and ligated. Clean-up was performed with SPRISelect beads before library preparation. DNA concentration was measured with the Qubit Fluorometer (Thermo Fisher Scientific) and Qubit HS Assay Kit. The biotinylation percentage was estimated using the Arima-HiC v2 QC beads.

***Hi-C library preparation and sequencing***

Biotinylated DNA constructs were fragmented using a Covaris E220 sonicator and size selected to 400–600 bp using SPRISelect beads. DNA was enriched with Arima-HiC v2 kit Enrichment beads. End repair, A-tailing, and adapter ligation were carried out with the NEBNext Ultra II DNA Library Prep Kit (New England Biolabs), following a modified protocol where library preparation occurs while DNA remains bound to the Enrichment beads. Library amplification was performed using KAPA HiFi HotStart mix and a custom Unique Dual Index (UDI) barcode set (Integrated DNA Technologies). Depending on sample concentration and biotinylation percentage determined at the crosslinking stage, libraries were amplified with 10–16 PCR cycles. Post-PCR clean-up was performed with SPRISelect beads. Libraries were quantified using the AccuClear Ultra High Sensitivity dsDNA Standards Assay Kit (Biotium) and a FLUOstar Omega plate reader (BMG Labtech).

Prior to sequencing, libraries were normalised to 10 ng/μL. Normalised libraries were quantified again to create equimolar and/or weighted 2.8 nM pools. Pool concentrations were checked using the Agilent 4200 TapeStation (Agilent) with High Sensitivity D500 reagents before sequencing. Sequencing was performed using paired-end 150 bp reads on the Illumina NovaSeq 6000.

### Genome assembly

Prior to assembly of the PacBio HiFi reads, a database of *k*-mer counts ( *k* = 31) was generated from the filtered reads using FastK. GenomeScope2 ( Ranallo-Benavidez *et al.*, 2020) was used to analyse the *k*-mer frequency distributions, providing estimates of genome size, heterozygosity, and repeat content.

The HiFi reads were assembled using Hifiasm ( Cheng *et al.*, 2021) with the --primary option. Haplotypic duplications were identified and removed using purge_dups ( Guan *et al.*, 2020). The Hi-C reads ( Rao *et al.*, 2014) were mapped to the primary contigs using bwa-mem2 ( Vasimuddin *et al.*, 2019), and the contigs were scaffolded in YaHS ( Zhou *et al.*, 2023) with the --break option for handling potential misassemblies. The scaffolded assemblies were evaluated using Gfastats ( Formenti *et al.*, 2022), BUSCO ( Manni *et al.*, 2021) and MerquryFK ( Rhie *et al.*, 2020).

The mitochondrial genome was assembled using MitoHiFi ( Uliano-Silva *et al.*, 2023).

### Assembly curation

The assembly was decontaminated using the Assembly Screen for Cobionts and Contaminants ( ASCC) pipeline. TreeVal was used to generate the flat files and maps for use in curation. Manual curation was conducted primarily in PretextView and HiGlass ( Kerpedjiev *et al.*, 2018). Scaffolds were visually inspected and corrected as described by Howe *et al*. (2021). Manual corrections included ten breaks, 35 joins, and removal of three haplotypic duplications. This reduced the scaffold count by 94.0%, increased the scaffold N50 by 41.5%, and reduced the total assembly length by 9.6%. The curation process is described at https://gitlab.com/wtsi-grit/rapid-curation . PretextSnapshot was used to generate a Hi-C contact map of the final assembly.

### Assembly quality assessment

The MerquryFK tool ( Rhie *et al.*, 2020) was run in a Singularity container ( Kurtzer *et al.*, 2017) to evaluate *k*-mer completeness and assembly quality for the primary and alternate haplotypes using the *k*-mer database ( *k* = 31) computed prior to genome assembly. The analysis outputs included assembly QV scores and completeness statistics.

The genome was analysed using the BlobToolKit pipeline, a Nextflow implementation of the earlier Snakemake version ( Challis *et al.*, 2020). The pipeline aligns PacBio reads using minimap2 ( Li, 2018) and SAMtools ( Danecek *et al.*, 2021) to generate coverage tracks. It runs BUSCO ( Manni *et al.*, 2021) using lineages identified from the NCBI Taxonomy ( Schoch *et al.*, 2020). For the three domain-level lineages, BUSCO genes are aligned to the UniProt Reference Proteomes database ( Bateman *et al.*, 2023) using DIAMOND blastp ( Buchfink *et al.*, 2021). The genome is divided into chunks based on the density of BUSCO genes from the closest taxonomic lineage, and each chunk is aligned to the UniProt Reference Proteomes database with DIAMOND blastx. Sequences without hits are chunked using seqtk and aligned to the NT database with blastn ( Altschul *et al.*, 1990). The BlobToolKit suite consolidates all outputs into a blobdir for visualisation. The BlobToolKit pipeline was developed using nf-core tooling ( Ewels *et al.*, 2020) and MultiQC ( Ewels *et al.*, 2016), with containerisation through Docker ( Merkel, 2014) and Singularity ( Kurtzer *et al.*, 2017).

## Genome sequence report

### Sequence data

**Figure 2.**
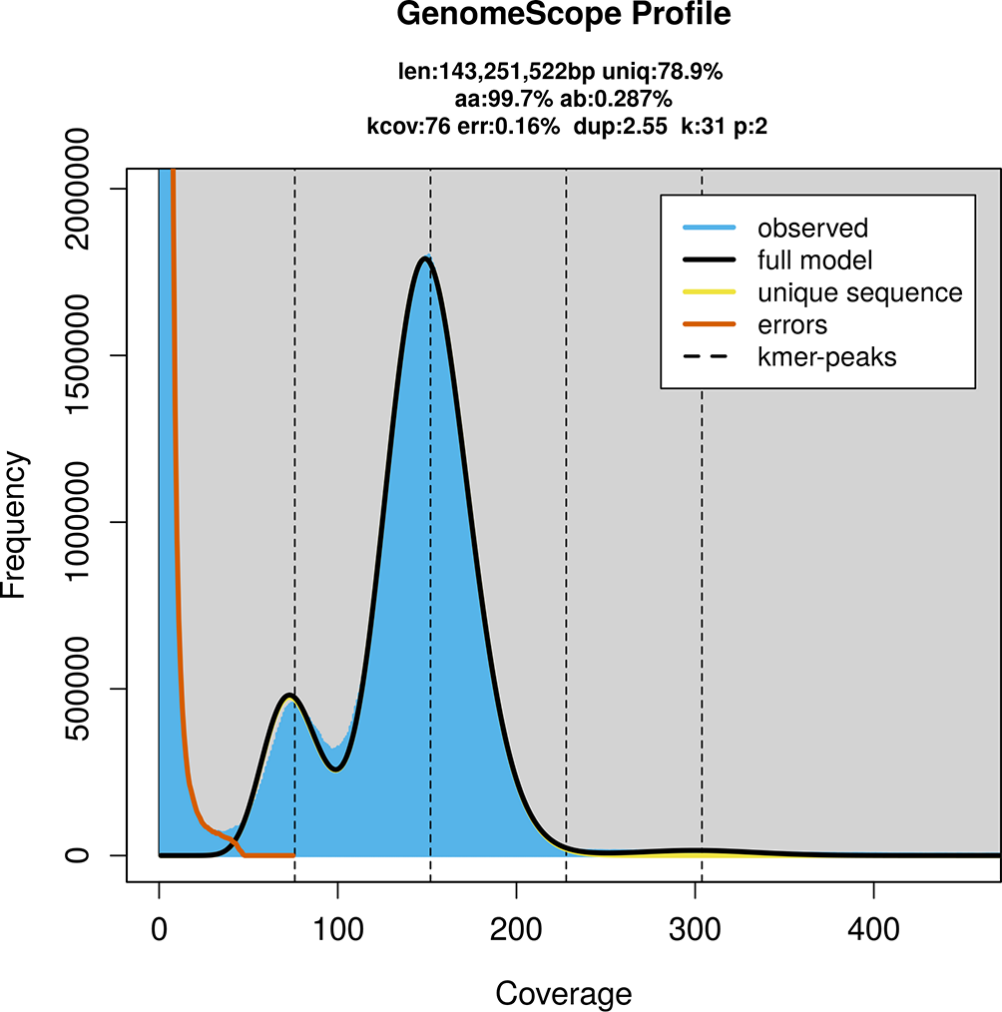
Frequency distribution of *k*-mers generated using GenomeScope2. The plot shows observed and modelled *k*-mer spectra, providing estimates of genome size, heterozygosity, and repeat content based on unassembled sequencing reads.

PacBio sequencing of the *Hololepta plana* specimen generated 24.50 Gb (gigabases) from 2.46 million reads, which were used to assemble the genome. GenomeScope2.0 analysis estimated the haploid genome size at 143.25 Mb, with a heterozygosity of 0.29% and repeat content of 21.12% ( Figure 2). These estimates guided expectations for the assembly. Based on the estimated genome size, the sequencing data provided approximately 152× coverage. Hi-C sequencing produced 98.20 Gb from 325.17 million reads, which were used to scaffold the assembly. Table 1 summarises the specimen and sequencing details.

**Table 1.** Specimen and sequencing data for *hololepta plana* (BioProject PRJEB64074).

Platform	PacBio HiFi	Hi-C
**ToLID**	icHolPlan1	icHolPlan1
**Specimen ID**	NHMUK014436789	NHMUK014436789
**BioSample (source individual)**	SAMEA111458819	SAMEA111458819
**BioSample (tissue)**	SAMEA111458840	SAMEA111458851
**Tissue**	head and thorax	abdomen
**Instrument**	Sequel IIe	Illumina NovaSeq 6000
**Run accessions**	ERR11673234	ERR11679387
**Read count total**	2.46 million	325.17 million
**Base count total**	24.50 Gb	98.20 Gb

### Assembly statistics

The primary haplotype was assembled, and contigs corresponding to an alternate haplotype were also deposited in INSDC databases. The final assembly has a total length of 139.51 Mb in 45 scaffolds, with 100 gaps, and a scaffold N50 of 14.29 Mb ( Table 2).

**Table 2.** Genome assembly data for *hololepta plana.*

Genome assembly	Primary assembly
**Assembly name**	icHolPlan1.2
**Assembly accession**	GCA_963695495.2
**Alternate haplotype accession**	GCA_963700325.2
**Assembly level**	chromosome
**Span (Mb)**	139.51
**Number of chromosomes**	12
**Number of contigs**	145
**Contig N50**	2.2 Mb
**Number of scaffolds**	45
**Scaffold N50**	14.29 Mb
**Sex chromosomes**	X
**Organelles**	Mitochondrion: 17.73 kb

**Figure 3.**
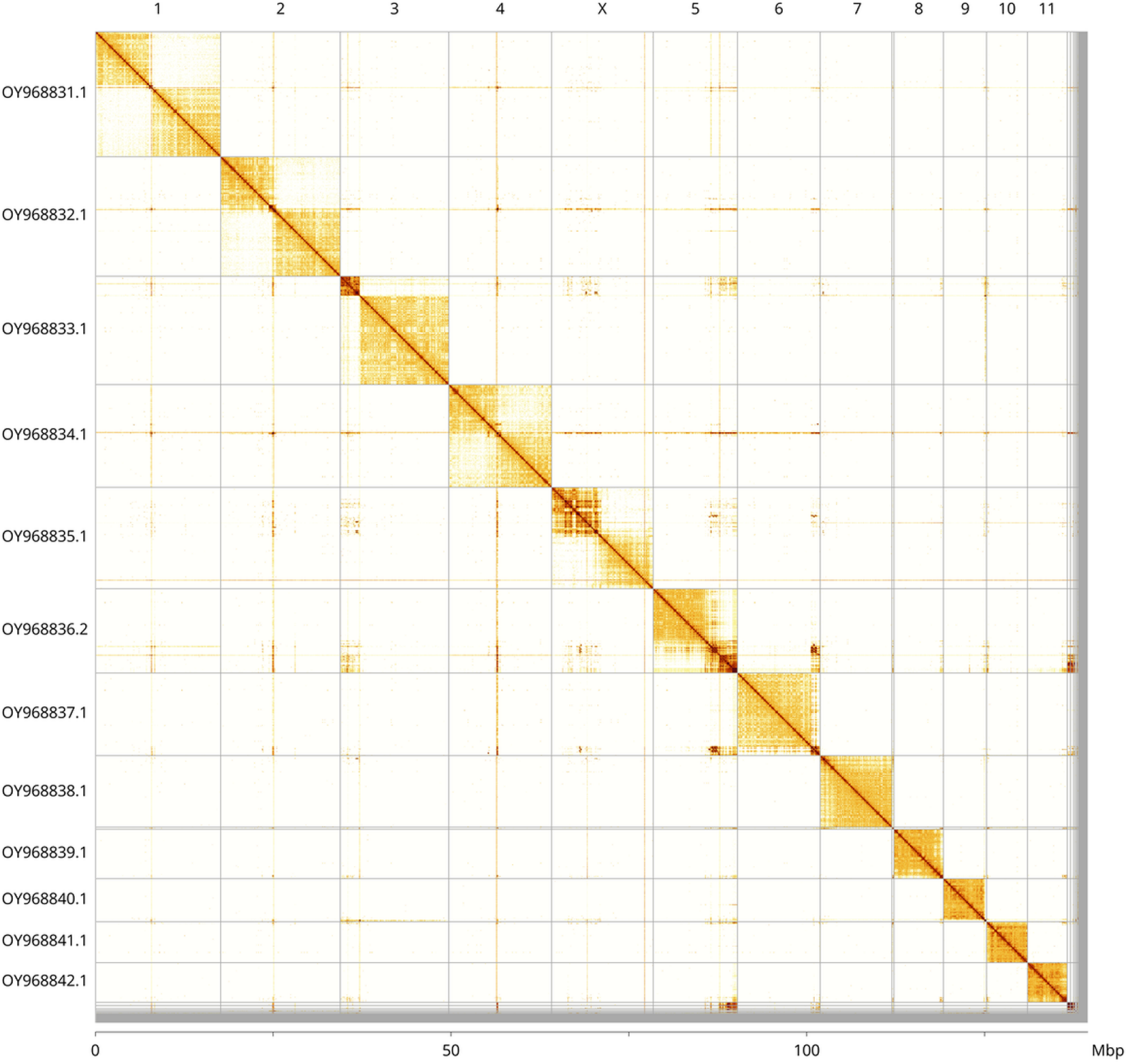
Hi-C contact map of the *Hololepta plana* genome assembly. Assembled chromosomes are shown in order of size and labelled along the axes, with a megabase scale shown below. The plot was generated using PretextSnapshot.

Most of the assembly sequence (97.93%) was assigned to 12 chromosomal-level scaffolds, representing 11 autosomes and the X sex chromosome. These chromosome-level scaffolds, confirmed by Hi-C data, are named according to size ( Figure 3; Table 3). X chromosome identified based on synteny with the genome of *Leptodirus hochenwartii* (GCA_947310635.1) ( Delić *et al.*, 2025).

**Table 3.** Chromosomal pseudomolecules in the primary genome assembly of *Hololepta plana* icHolPlan1.

INSDC accession	Molecule	Length (Mb)	GC%
OY968831.1	1	17.62	34.50
OY968832.1	2	16.83	35.50
OY968833.1	3	15.23	36
OY968834.1	4	14.46	35.50
OY968836.2	5	11.85	38
OY968837.1	6	11.63	36
OY968838.1	7	10.37	38
OY968839.1	8	6.98	39.50
OY968840.1	9	6.04	41
OY968841.1	10	5.74	41.50
OY968842.1	11	5.58	41.50
OY968835.1	X	14.29	35.50

The mitochondrial genome was also assembled (length 17.73 kb, OY968843.1). This sequence is included as a contig in the multifasta file of the genome submission and as a standalone record.

### Assembly quality metrics

**Figure 4.**
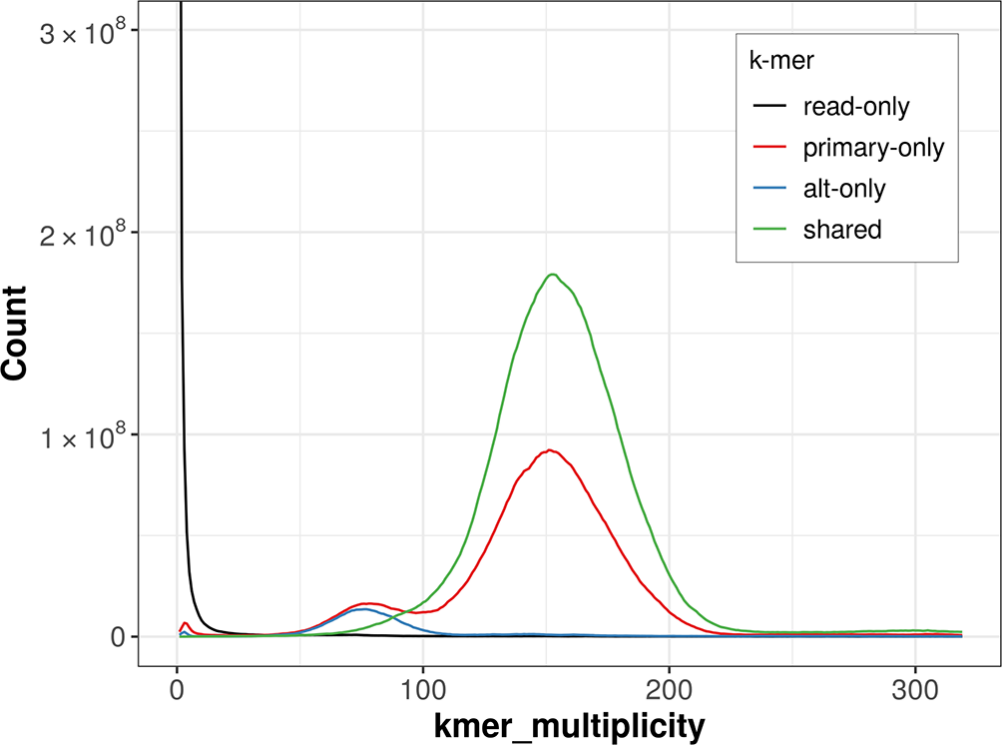
Evaluation of *k*-mer completeness using MerquryFK. This plot illustrates the recovery of *k*-mers from the original read data in the final assemblies. The horizontal axis represents *k*-mer multiplicity, and the vertical axis shows the number of *k*-mers. The black curve represents *k*-mers that appear in the reads but are not assembled. The green curve corresponds to *k*-mers shared by both haplotypes, and the red and blue curves show *k*-mers found only in one of the haplotypes.

The combined primary and alternate assemblies achieve an estimated QV of 55.1. The *k*-mer completeness is 93.62% for the primary assembly, 64.96% for the alternate haplotype, and 99.13% for the combined assemblies ( Figure 4).

**Figure 5.**
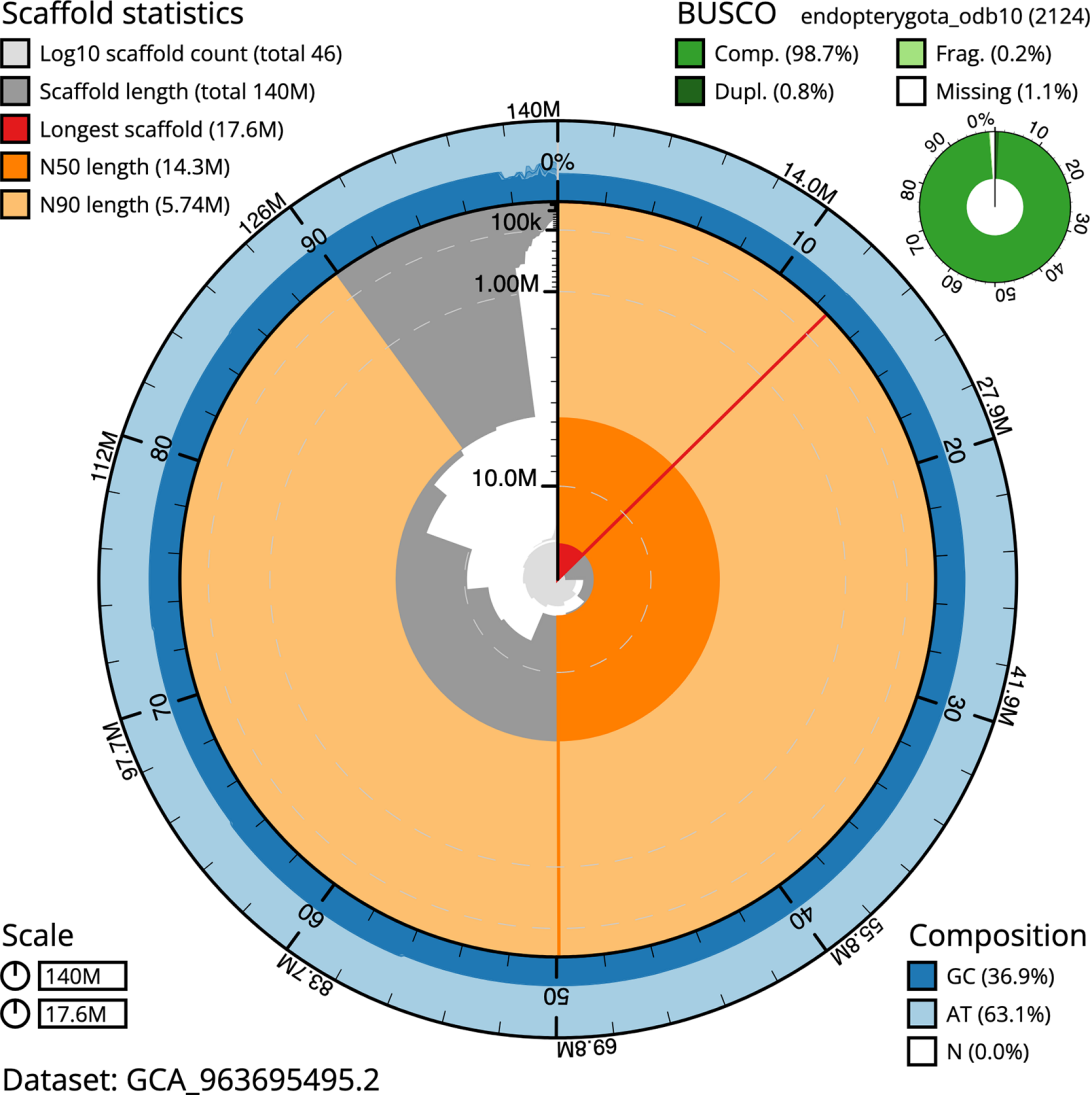
Assembly metrics for icHolPlan1.2. The BlobToolKit snail plot provides an overview of assembly metrics and BUSCO gene completeness. The circumference represents the length of the whole genome sequence, and the main plot is divided into 1 000 bins around the circumference. The outermost blue tracks display the distribution of GC, AT, and N percentages across the bins. Scaffolds are arranged clockwise from longest to shortest and are depicted in dark grey. The longest scaffold is indicated by the red arc, and the deeper orange and pale orange arcs represent the N50 and N90 lengths. A light grey spiral at the centre shows the cumulative scaffold count on a logarithmic scale. A summary of complete, fragmented, duplicated, and missing BUSCO genes in the endopterygota_odb10 set is presented at the top right. An interactive version of this figure can be accessed on the BlobToolKit viewer.

**Figure 6.**
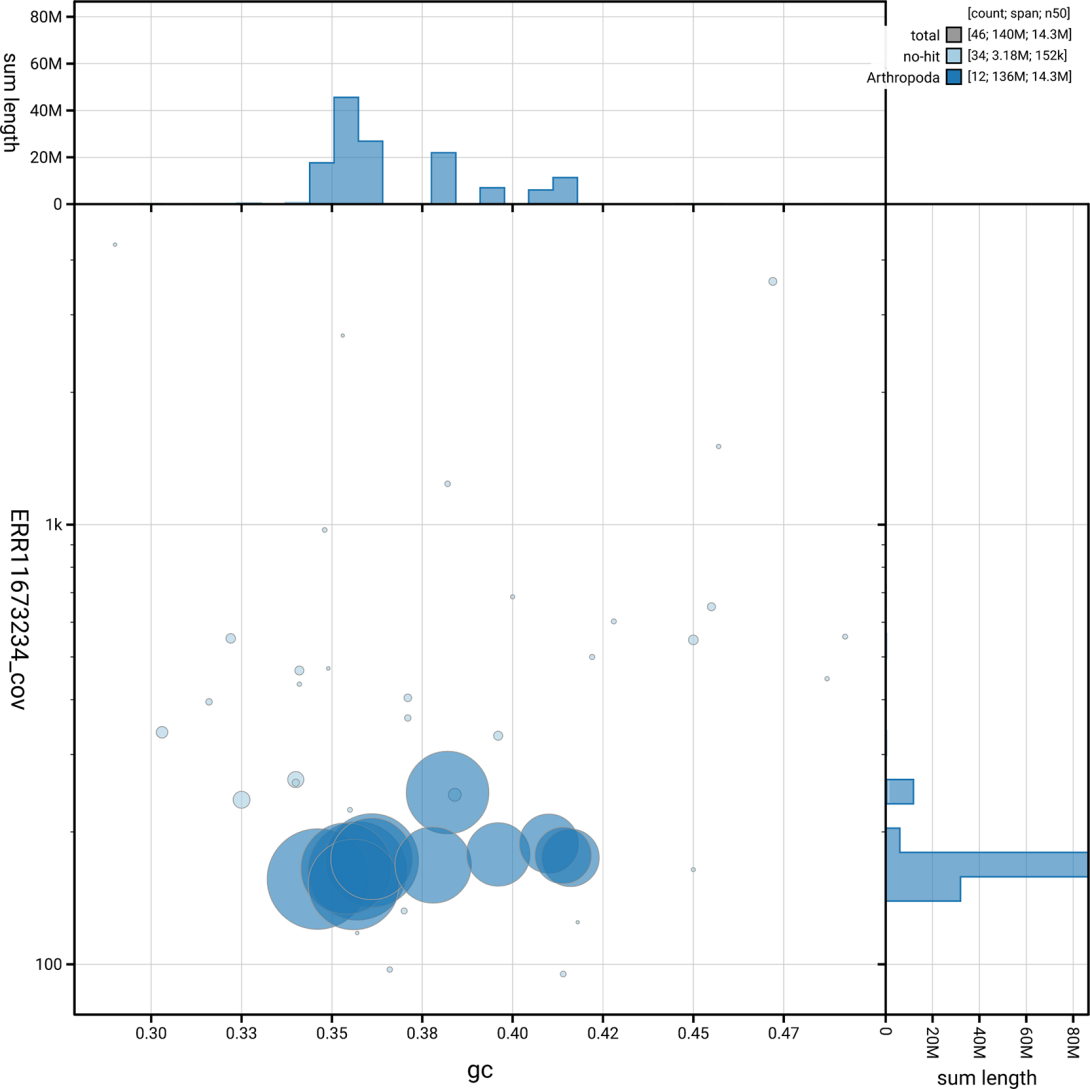
BlobToolKit blob plot for icHolPlan1.2. The plot shows base coverage (vertical axis) and GC content (horizontal axis). The circles represent scaffolds, with the size proportional to scaffold length and the colour representing phylum membership. The histograms along the axes display the total length of sequences distributed across different levels of coverage and GC content. An interactive version of this figure is available on the BlobToolKit viewer.

BUSCO v.6.0.0 analysis using the endopterygota_odb10 reference set ( *n* = 2 124) identified 98.7% of the expected gene set (single = 97.9%, duplicated = 0.8%). The snail plot in Figure 5 summarises the scaffold length distribution and other assembly statistics for the primary assembly. The blob plot in Figure 6 shows the distribution of scaffolds by GC proportion and coverage.

Table 4 lists the assembly metric benchmarks adapted from Rhie *et al.* (2021) and the Earth BioGenome Project Report on Assembly Standards January 2026. The EBP metric, calculated for the primary assembly, is **6.C.Q54**, meeting the recommended reference standard.

**Table 4.** Earth Biogenome Project summary metrics for the *Hololepta plana* assembly.

Measure	Value	Benchmark
EBP summary (primary)	6.C.Q54	6.C.Q40
Contig N50 length	2.20 Mb	≥ 1 Mb
Scaffold N50 length	14.29 Mb	= chromosome N50
Consensus quality (QV)	Primary: 54.1; alternate: 55.9; combined: 55.1	≥ 40
*k*-mer completeness	Primary: 93.62%; alternate: 64.96%; combined: 99.13%	≥ 95%
BUSCO	C:98.7% [S:97.9%, D:0.8%], F:0.2%, M:1.1%, n:2 124	S > 90%; D < 5%
Percentage of assembly assigned to chromosomes	97.93%	≥ 90%

**Notes:** The EBP summary uses log10(Contig N50); chromosome-level (C) or log10(Scaffold N50); Q (Merqury QV). BUSCO: C = complete; S = single-copy; D = duplicated; F = fragmented; M = missing; n = orthologues

**Table 5.** Software versions and sources used for *Hololepta plana.*

Software	Version	Source
BLAST	2.14.0	ftp://ftp.ncbi.nlm.nih.gov/blast/executables/blast+/
BlobToolKit	4.4.6	https://github.com/blobtoolkit/blobtoolkit
BUSCO	6.0.0	https://gitlab.com/ezlab/busco
bwa-mem2	2.2.1	https://github.com/bwa-mem2/bwa-mem2
DIAMOND	2.1.8	https://github.com/bbuchfink/diamond
fasta_windows	0.2.4	https://github.com/tolkit/fasta_windows
FastK	1.1	https://github.com/thegenemyers/FASTK
GenomeScope2.0	2.0.1	https://github.com/tbenavi1/genomescope2.0
Gfastats	1.3.6	https://github.com/vgl-hub/gfastats
Hifiasm	0.19.5-r587	https://github.com/chhylp123/hifiasm
HiGlass	1.13.4	https://github.com/higlass/higlass
MerquryFK	1.1.2	https://github.com/thegenemyers/MERQURY.FK
Minimap2	2.28-r1209	https://github.com/lh3/minimap2
MitoHiFi	3	https://github.com/marcelauliano/MitoHiFi
MultiQC	1.14; 1.17 and 1.18	https://github.com/MultiQC/MultiQC
Nextflow	24.10.4	https://github.com/nextflow-io/nextflow
PretextSnapshot	0.0.5	https://github.com/sanger-tol/PretextSnapshot
PretextView	1.0.3	https://github.com/sanger-tol/PretextView
purge_dups	1.2.5	https://github.com/dfguan/purge_dups
samtools	1.21	https://github.com/samtools/samtools
sanger-tol/ascc	0.1.0	https://github.com/sanger-tol/ascc
sanger-tol/blobtoolkit	v0.9.0	https://github.com/sanger-tol/blobtoolkit
sanger-tol/curationpretext	1.4.2	https://github.com/sanger-tol/curationpretext
Seqtk	1.3	https://github.com/lh3/seqtk
Singularity	3.9.0	https://github.com/sylabs/singularity
TreeVal	1.4.0	https://github.com/sanger-tol/treeval
YaHS	1.2a.2	https://github.com/c-zhou/yahs

## Data Availability

European Nucleotide Archive: Hololepta plana. Accession number PRJEB64074. The genome sequence is released openly for reuse. The *Hololepta plana* genome sequencing initiative is part of the Darwin Tree of Life Project (PRJEB40665) and the Sanger Institute Tree of Life Programme (PRJEB43745). All raw sequence data and the assembly have been deposited in INSDC databases. The genome will be annotated using available RNA-Seq data and presented through the Ensembl pipeline at the European Bioinformatics Institute. Raw data and assembly accession identifiers are reported in Tables 1 and 2. Production code used in genome assembly at the WSI Tree of Life is available at https://github.com/sanger-tol . Table 5 lists software versions used in this study.

## Author information

Contributors are listed at the following links:

• Members of the Natural History Museum Genome Acquisition Lab
• Members of the Darwin Tree of Life Barcoding collective
• Members of the Wellcome Sanger Institute Tree of Life Management, Samples and Laboratory team
• Members of Wellcome Sanger Institute Scientific Operations – Sequencing Operations
• Members of the Wellcome Sanger Institute Tree of Life Core Informatics team
• Members of the Tree of Life Core Informatics collective
• Members of the Darwin Tree of Life Consortium

## Wellcome sanger institute – legal and governance

The materials that have contributed to this genome note have been supplied by a Darwin Tree of Life Partner. The submission of materials by a Darwin Tree of Life Partner is subject to the **‘Darwin Tree of Life Project Sampling Code of Practice’**, which can be found in full on the Darwin Tree of Life website. By agreeing with and signing up to the Sampling Code of Practice, the Darwin Tree of Life Partner agrees they will meet the legal and ethical requirements and standards set out within this document in respect of all samples acquired for, and supplied to, the Darwin Tree of Life Project. Further, the Wellcome Sanger Institute employs a process whereby due diligence is carried out proportionate to the nature of the materials themselves, and the circumstances under which they have been/are to be collected and provided for use. The purpose of this is to address and mitigate any potential legal and/or ethical implications of receipt and use of the materials as part of the research project, and to ensure that in doing so we align with best practice wherever possible. The overarching areas of consideration are:

• Ethical review of provenance and sourcing of the material
• Legality of collection, transfer and use (national and international)

Each transfer of samples is further undertaken according to a Research Collaboration Agreement or Material Transfer Agreement entered into by the Darwin Tree of Life Partner, Genome Research Limited (operating as the Wellcome Sanger Institute), and in some circumstances, other Darwin Tree of Life collaborators.
