# Supplementary material for: The genome sequence of the flat clown beetle, Hololepta plana (Sulzer, 1776) (Coleoptera: Histeridae)
Source: Wellcome Open Res. 2026 May 18;11:289. [Version 1] doi: 10.12688/wellcomeopenres.26588.1 (PMC13237540; doi:10.12688/wellcomeopenres.26588.1)
Supplement: Peer review history

# Reviewer report: Reviewer response for version 1

**DOI:** 10.21956/wellcomeopenres.29281.r156252

**Published:** 2026-06-04

**Authors:** Henrik Lantz

**Affiliations:** 1 SciLifeLab, Uppsala University, Uppsala, Sweden

This manuscript presents the genome sequence of the flat clown beetle, Hololepta plana, assembled as a part of the Darwin Tree of Life project. The species is rare in Britain, and was only first collected there in 2009, making experts think the species most likely is a recent immigrant. The species is large compared to closely related species and has specific morphological features which makes it a distinct species in the family Histeridae.

 The assembled genome is of high quality and can be used to determine the origin of the British population and how it is related to other populations in continental Europe. 97.93% of the assembly (a high number) is scaffolded into 12 chromosomal pseudomolecules, including the sex determination X chromosome. BUSCO score is also very high (98.7%) and the assembly easily meets all of the Earth Biogenome Project criteria except possibly the scaffold N50 value as not 100% of the scaffolds are assigned to chromosomes, but that difference is negligible.

 Methods follow a high international standard and are in general well-described, except for some few cases commented below.

 1. Figure 1, and text: The specimen used has been documented by a picture which is good, and the text explains how tissues were dissected and used for DNA extraction and barcoding, but there is no information if any material has been saved in a museum and/or biobank. The morphology of the species seems distinctive enough that it should be possible to determine the identity of the specimen from the photo, but I would prefer that a high quality photo is preserved in a Natural History Museum and also any remaining tissue, if available.

 2. Figure 3. The HiC contact map shows some remaining contacts between different scaffolds, e.g., between OY968836.2 and OY968837.1. This is most likely caused by repeats and not really something that needs to be corrected, but it would be good to have the results of the curation discussed more in detail in the text.

 3. The BlobToolKit plot in Figure 6 shows some small blobs outside of the main cluster with no hits in the database, hinting at remaining contamination. While this is unlikely to cause any big problems in downstream analyses, I would prefer if the results of the de-contamination would be discussed in the text.

 4. Simply stating that the mitochondrion was assembled using MitoHiFi is not enough to ensure reproducibility. Settings should be mentioned, or even better, scripts should be supplied in a GitHub repo.

Are sufficient details of methods and materials provided to allow replication by others?

Partly

Is the rationale for creating the dataset(s) clearly described?

Yes

Are the datasets clearly presented in a useable and accessible format?

Yes

Are the protocols appropriate and is the work technically sound?

Yes

Reviewer Expertise:

Genome assembly, genome annotation, phylogenetics, taxonomy, systematics.

I confirm that I have read this submission and believe that I have an appropriate level of expertise to confirm that it is of an acceptable scientific standard.

# Reviewer report: Reviewer response for version 1

**DOI:** 10.21956/wellcomeopenres.29281.r156249

**Published:** 2026-06-03

**Authors:** Rajib Deb

**Affiliations:** 1 ICAR-National Research Centre, Guwahat, Assam, India

The rationale for generating the genome dataset is clearly explained and biologically justified.

 The sequencing, assembly, and curation protocols are appropriate and technically sound, using current standard methodologies and software tools.

 Methods and materials are described in sufficient detail to enable reproducibility, including software versions, sequencing platforms, and accession numbers

 The genome assembly is of high quality, with strong BUSCO completeness and chromosome-level scaffolding statistics. The datasets are clearly presented and openly accessible through public repositories, making them usable for future research.

 However, following issues may be resolved:

- The manuscript could briefly clarify whether any residual unplaced scaffolds contain repetitive or low-complexity regions.
- A short statement on expected future genome annotation outcomes or comparative genomic applications would further enhance the utility of the note.
- Figure legends are generally clear, but adding a brief interpretation of the Hi-C contact map quality in the text would improve accessibility for non-specialist readers.
- Minor typographical and formatting consistency checks (e.g., spacing around percentages and symbols) may improve readability.

Are sufficient details of methods and materials provided to allow replication by others?

Yes

Is the rationale for creating the dataset(s) clearly described?

Yes

Are the datasets clearly presented in a useable and accessible format?

Yes

Are the protocols appropriate and is the work technically sound?

Yes

Reviewer Expertise:

Genomics

I confirm that I have read this submission and believe that I have an appropriate level of expertise to confirm that it is of an acceptable scientific standard.
